# Resistance Exercise Training on Musculoskeletal, Metabolic and Psychological Health in Sedentary Office Workers – Systematic Review and Meta-analysis

**DOI:** 10.1007/s10926-025-10273-8

**Published:** 2025-02-14

**Authors:** Baskaran Chandrasekaran, Kalyana Chakravarthy Bairapareddy, Chythra R. Rao

**Affiliations:** 1 Department of Exercise and Sports Sciences, Manipal College of Health Professions, Manipal Academy of Higher Education, Manipal, Karnataka 576104 India; 2 Department of Physiotherapy, College of Health Sciences, University of Sharjah, Sharjah, United Arab Emirates; 3 Department of Community Medicine, Kasturba Medical College, Manipal Academy of Higher Education, Manipal, Karnataka 576104 India

**Keywords:** Strength training, Cardiometabolic risk, Pain, Office worker, Sedentary, Worker

## Abstract

**Purpose:**

To consolidate the emerging evidence on the effectiveness of resistance training (RT) in reducing the health risks among sedentary office workers.

**Methods:**

Four electronic databases were searched for evidence from its inception till september 20, 2024. Studies were included if they examined any form of RT program targeting musculoskeletal, metabolic, or psychological health outcomes in office workers aged 18 years or older using PICOS criteria (Population – office workers, Intervention – RT program, Comparison – placebo or sham control and Outcomes – musculoskeletal, cardiometabolic and psychological health variables). Two reviewers independently screened the studies for risk of bias and assessed the certainty of the evidence.

**Results:**

Out of 60 identified studies, 17 studies were eligible for narrative synthesis, and 16 were included in the meta-analysis. Modest reductions in neck (SMD = -1.76, I^2^ = 88%, p < 0.00001) and shoulder discomfort (SMD = -13.29, I^2^ = 91%, p < 0.00001), while marginal improvement in shoulder (SMD = 4.13, I^2^ = 99%, p = 0.03) and neck extensor muscle strength (SMD = 9.07, I^2^ = 9%, p < 0.00001). The cardiometabolic and mental health risk markers remain unaltered. High uncertainty of evidence was observed due to high heterogeneity, risk of bias, inconsistency and publication bias.

**Conclusion:**

Limited evidence demonstrate supervised RT programs of any dose has a potential to improve muscular strength and discomfort while potential cardiometabolic and mental health risk outcomes remain unaltered. However, more high-quality research trials are needed to understand the effects of RT on health benefits.

**Supplementary Information:**

The online version contains supplementary material available at 10.1007/s10926-025-10273-8.

## Introduction

Non-communicable diseases, including cardiometabolic, musculoskeletal, and psychological disorders, are among the leading causes of morbidity and premature mortality in adults worldwide [1]. Population-based studies clustering risk factors have identified physical inactivity and excessive sedentary time as significant contributors to non-communicable diseases (NCDs), alongside other factors such as low fruit consumption, smoking, and alcohol use [2, 3]. Sedentary behavior, defined as activities during waking hours with low energy expenditure (less than 1.5 metabolic equivalents), has become a major public health concern in recent years [4]. As newer technologies are continuously adapted, the office workers spent significant proportion of their office workers in seated postures [5]. Prolonged sedentary behavior, independent of physical inactivity (defined as not meeting the public guideline of 150 min of moderate-to-vigorous activity per week), is associated with static muscle postures, eventually leading to musculoskeletal injury and pain [6]. Musculoskeletal discomfort may lead to limited physical mobility leading to poor cardiometabolic health and psychological outcomes [7]. There is a growing interest in exploring resistance exercise that offer favorable effects not only on musculoskeletal health outcomes but also the prevention from cardiometabolic risk [8].

Previous systematic reviews consistently highlight the modest effects of workplace physical activity and sedentary behavior interventions on potential cardiometabolic and musculoskeletal benefits among office workers [9–12]. While Reed (2017) reported favorable changes in most cardiometabolic markers [9], Mulchandani (2019) observed only modest but significant reductions in body mass and waist circumference, with no improvements in glucose, blood pressure, or lipid levels, even with multicomponent interventions [10]. Although these reviews suggest that interventions of sufficient intensity (moderate-to-vigorous) could yield favorable changes in cardiometabolic disease risk, a recent review found that adding treadmill workstations did not produce significant improvements in cardiometabolic risk markers [13]. This underscores the need for a feasible yet effective lifestyle intervention that can be seamlessly integrated into the daily routines of sedentary office workers.

Resistance training (RT), an exercise modality that progressively increases external resistance in line with an individual’s growing strength, has gained prominence for its effectiveness in preventing musculoskeletal disorders in non-athlete populations [14]. Further, RT is now viewed as a promising countermeasure to vascular dysfunction, postprandial glycemia, and lipidemia associated with prolonged sitting in office workers [15–18]. Moreover, RT is an accessible and feasible strategy that can be incorporated into the workstation without the need for specialized equipment [19]. Recent empirical studies have explored the favorable effects of RT on musculoskeletal and cardiometabolic health outcomes in office workers during work hours and non-work hours and the findings remain unconsolidated [15–18]. To our knowledge, there are no systematic reviews that have consolidated the contemporary evidence that has explored the independent health benefits of resistance exercise training among sedentary office workers.

Thus, the present systematic review and meta-analysis aimed to synthesize the current literature on the effects of resistance exercise training on musculoskeletal and related health outcomes among sedentary office workers. The findings may enhance the public health guidelines to implement the RT program in contextual settings.

## Methods

The present systematic review is reported as per the guidelines laid by the Preferred Reporting Items for Systematic Reviews and Meta-Analyses (PRISMA) [20]. The PRISMA checklist is attached as supplementary file S1.

## Search Strategy

The search strategy was built using the following keywords: resistance exercise or strength training, desk-based or sedentary office workers, and musculoskeletal, cardiometabolic and psychological health outcomes. We used following strategy to explore the efficacy of RT program among office workers: “Strength training” OR “resistance exercise” OR “resistance exercise training” OR dumbbell OR “barbell training” OR “kettle bell” OR “weight training” OR “calisthenics” OR “resistance bands” AND “office workers” OR “desk-based office workers” OR “desk-based job” OR “sedentary job” OR “white-collar workers” OR “knowledge workers” OR “corporate employees”. We administered the search strategy in four electronic databases of peer-reviewed journals, including Embase, Cumulative Index of Nursing and Allied Health Literature (CINAHL), Scopu,s and Web of Science. Additionally, complementary strategies were utilized during the full-text review. These included backward and forward citation tracking, as well as the use of artificial intelligence tools like Research Rabbit and SciSpace to retrieve secondary references alongside primary references. The search was administered from the inception of data sources till 27th April 2024. The search was updated on 20th September 2024. The search strategy was provided as a supplementary file S1.

## Eligibility Criteria

We have framed our eligibility criteria based on Population, Intervention, Comparison/control and Study Type (PICOS) criteria as endorsed by Cochrane Collaboration and PRISMA guidelines [21]. To be included, the study should satisfy the following criteria outlined in Table 1 below.

**Table 1:** Eligibility criteria of the to be included studies for the review using PICOS strategy

PICOS parameters	Eligibility criteria
**P**opulation	• Studies involved adults > 18 years and older age • Sedentary and physically inactive office workers • With or without cardiometabolic, musculoskeletal and mental health risk
**I**ntervention	• Resistance training (RT) administered as a primary intervention • Administered as a continuous bout or breaks • Implemented in workplace context (during work and non-working hours) • Involved any type of RT programs (ranging from body-supported resistance exercise to equipment-based RT programs—dumbbell, resistance bands and tubes, hydraulic machines, and pulleys)
**C**omparison	• RT group compared against a control or a parallel group which has not received RT • Single group with control period and adequate wash-out
**O**utcomes	• Musculoskeletal outcomes: pain/discomfort, muscular strength, muscular endurance, flexibility, agility • Cardiometabolic outcomes: blood pressure, body composition, aerobic fitness, biochemistry parameters, vascular functions, autonomic stability • Mental health: depression, anxiety, stress
**S**tudy design	• Randomized and non-randomized controlled trials • Secondary analysis from randomized controlled trials
Additional criteria	• Published in English • Only full-text articles • No restriction on geographical locations and period of publication • When two different studies with duplicated data (redundant publication) were available, primary study was considered for the data extraction

RT: resistance training

## Study Selection

The studies from the databases were downloaded as.ris files and were imported to the EndNote online reference management software (EndNote, Clarivate), and duplicates were removed. After de-duplication, two authors (BC and KC) shared the folder with the studies and started sorting the studies based on the priori determined eligibility criteria mentioned above. Two authors (BC and KC) independently screened for the title, abstract and full text of the studies and met with mutual agreement on the inclusion of the studies. If the two authors could not reach a mutual agreement, a third author (CRR) was consulted to determine the inclusion of the article in the review.

## Data Extraction

A bespoke data sheet was prepared to extract succinate content from the studies included for the review using Microsoft Excel 2016 spreadsheet (Microsoft Office 16, USA). The primary author (BC) extracted the data and filled in the excel sheets with the following variables: author, year, study design, participant characteristics, context (non-work, work hours), RT program details (supervised or unsupervised, mode – resistance bands, body supported, free weights, frequency, duration, intensity – maximal voluntary contraction, one-repetition maximum, volume and progression of RT, intermittent, continuous), outcome measures (musculoskeletal outcomes: musculoskeletal pain/discomfort, pain sensitivity, muscle strength, mass and flexibility; cardiometabolic outcomes: blood pressure, body mass index, blood glucose and lipid profile and other inflammatory markers – interleukins, tumor necrosis factor, insulin like growth factor; mental health outcomes – depression, anxiety and stress, adverse events), and key findings. Further the mean differences of RT intervention and control group (Post – pre values) and standard deviations with the units of the variable were extracted by the primary author (BC) and cross-verified by the co-authors (KC & CRR). When the mean and standard deviation of the pre-post intervention and control groups were available only in graphs, the numerical data was extracted using Web Plot Digitizer (https://automeris.io/). For studies where the mean and standard deviation data were unavailable, attempts were made to contact the corresponding authors to obtain the required information. Studies were excluded from the analysis if the requested data could not be obtained.

## Risk of Bias Assessment

The risk of bias was assessed using updated Cochrane ROB-2 tool for randomized controlled trials and ROBINS-I tool for non-randomized controlled trials using Cochrane Systematic Review software (RevMan 5.4, Cochrane Collaboration, 2020). The included randomized controlled trials were judged for five biases under six domains: (a) randomization or sequence generation (selection bias); (b) allocation concealment (selection bias; (c) blinding (performance and detection bias); (d) incomplete outcome data (attrition bias); (e) selective reporting (reporting bias); and (f) other biases (group contamination, reactivity bias and adherence). Bias was scored as ‘high risk’, unclear risk’ and ‘’low risk’ based on the signaling questions. Two authors (BC & KC) independently assessed the risk of bias for each domain prior to the commencement of pooling of studies and data extraction.

## Data Analysis

Effects on musculoskeletal discomfort, strength and cardiometabolic health markers such as body fat percentages, aerobic capacity, systolic pressure, waist hip ratio and body mass index were carried out in Review Manager version 5.4 and guided by the chapter 10 of Cochrane Handbook (https://training.cochrane.org/handbook/current/chapter-10). The mean difference in the musculoskeletal, cardiometabolic and mental health outcomes of both RT trained, and control groups were determined from the baseline and the last follow-up alone was taken while the interim measurements were missed. Heterogeneity among the included studies was assessed using the Cochran’s Q test and quantified with the I^2^ statistic. I^2^ values were interpreted as follows: low heterogeneity (0–25%), moderate heterogeneity (26–50%), substantial heterogeneity (51–75%), and considerable heterogeneity (> 75%). Standardized mean difference (SMD) was used to quantify the effect size when the outcomes were quantified using different means of measurement and random effects model. Effect sizes were categorized as large, medium, small, or negligible when the standardized mean difference (SMD) exceeded 0.8, ranged from 0.5 to 0.8, ranged from 0.2 to 0.5, and was less than 0.2, respectively. The post values were subtracted from the pre-intervention or baseline values and were standardized to percentages. Mean differences, standard mean differences (Sect. 6.5.1.2) and standard deviations (Sect. 6.5.2.2) were calculated using the formula and guidelines outlined in Cochrane Handbook [22]. Egger’s test was carried out to test publication bias and if evident, trim-fill method was used to evaluate the impact of publication bias. Sensitivity analyses were carried out using leave out studies of low quality and different doses of RT when appropriate.

***Assessment of certainty of evidence:*** The overall quality of evidence was assessed using Grading of Recommendations Assessment, Development, and Evaluation (GRADE) criteria as reported in previous systematic review [23]. Using this criterion, the quality of evidence was categorized as very low, low, moderate and high certainty based on five domains: risk of bias (did not meet 50% of the risk of bias domains explained earlier), imprecision (too large confidence intervals or one study reporting the outcome), inconsistency (high statistical heterogeneity I^2^ > 75%), indirectness (poor generalizability based on eligibility criteria)and publication bias (deviation from protocol or large positive effects) [23]. The publication bias of the included studies was assessed by examining funnel plots and funnel plot asymmetry using Eggers linear regression tests whenever 10 or more studies were combined for a meta-analysis [24]. The visualization plots were visualized through RevMan 5.4 software.

## Results

### Study Selection

**Fig. 1:**
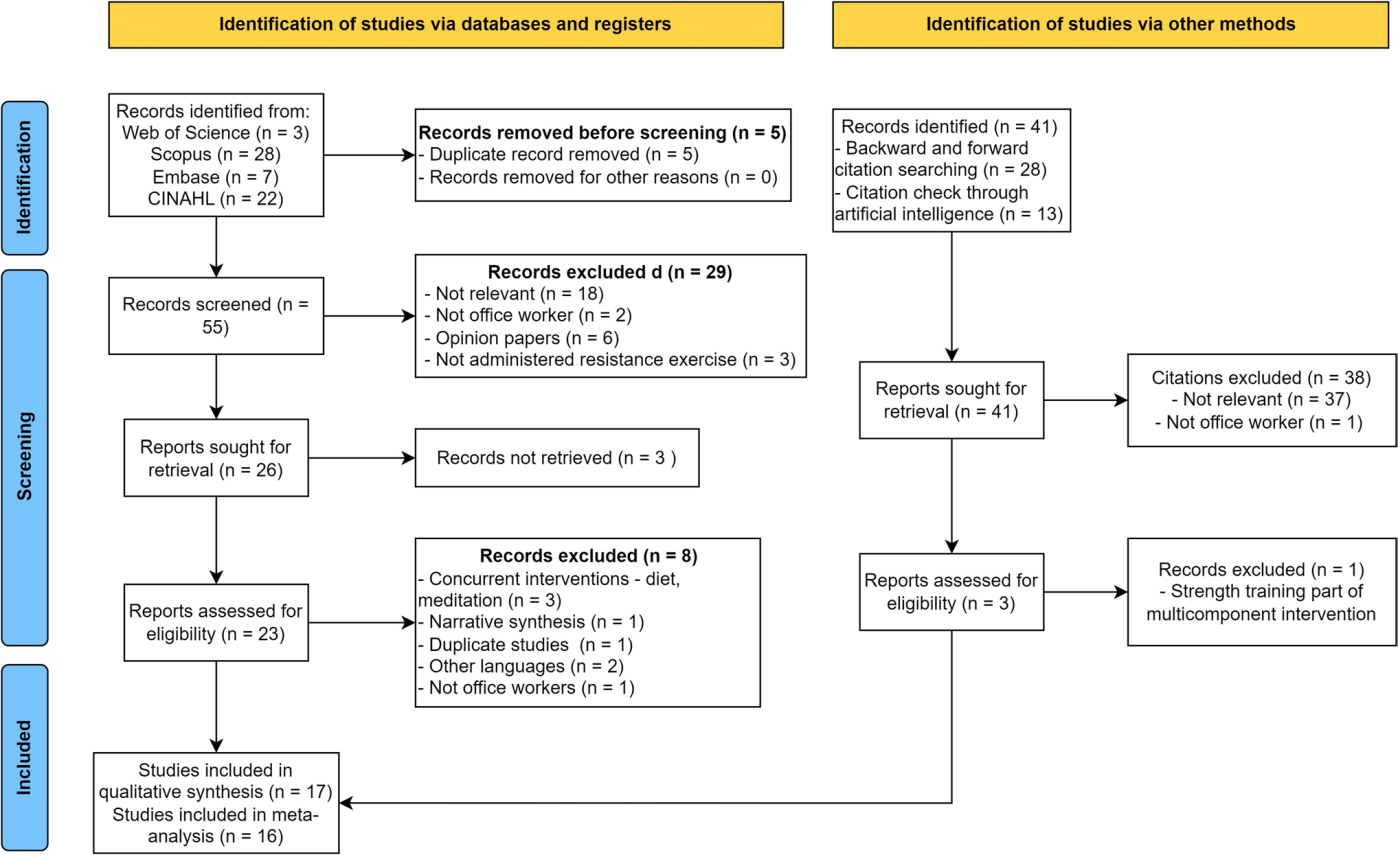
Screening and inclusion of the studies

The initial search yielded 60 studies from the four databases. After duplicates, 55 studies were available for title, abstract and full text screening. Following the abstract and full-text screening, 17 studies were included in the qualitative synthesis, and 16 studies in the quantitative synthesis. Since two studies analyzed the same data, only the data from primary study was retained for the analysis [25, 26]. Figure 1 depicts the screening and inclusion of the studies for the present systematic review.

## Characteristics of the Included Studies

Table 2 explains the population, interventions, outcomes, and the key findings of the included studies. A total of 17 studies were included with ten randomized controlled trials (59%) [15–18, 25–31], three experimental studies [18, 32, 33], two longitudinal studies [34, 35], one secondary analysis from RCTs [36] and one cross-over trial [37] with one trial reporting the findings of two trials (one cross-sectional and one randomized controlled trial) [29]. All the studies originated from high-income countries, with a majority from Denmark (n = 5/17, 29%) [16, 27, 30, 35], while few from the following countries: Australia [18, 36], Canada [17], Finland [25], Greece [32], Norway [37], Spain [29], Switzerland [34], Taiwan [28], Turkey [31], and USA [15, 33]. None of the included studies have originated from the low-middle income countries.

**Table 2:** Characteristics of the studies and their key findings

Author (year)	Study design	Country	Participant	Intervention	Outcome measure	Key findings
Andersen et al., 2008 [30]	C-RCT	Denmark	• 549 Denmark office workers • aged 45 years	• RT with dumbbells for neck and shoulders 2–3 sets of 10–15 repetitions • Randomized to one of the following: 1) SRT, 2) APE and 3) REF with counseling	• MVC using Bofors dynamometer • Self-reported neck and shoulder discomfort	• shoulder elevation strength ↑ 11% • Shoulder abduction strength ↑—SRT 12% and APE 10% • Neck pain ↓ 1.6 and 1.4 in SRT and APE respectively
Andersen et al., 2012 [16]	C-RCT	Denmark	• 449 employees (256 neck pain cases) • at least three on a 0–9 scale)	Four groups: (1) 60 min once per week, (2) 20 min thrice per week, (3) 9 min six days per week and (4) reference group	• Musculoskeletal symptoms – Nordic • Disabilities of Arm, Shoulder, Hand questionnaire • Self-reported muscle strength	• Adherence ↑ in 20 min and 9 min/day compared to 60 min/day • No diff in neck pain • Shoulder pain ↓ in the RT group • ↑ in 10-RM in a 60 min group compared to 7 min/day for six days
Dalager et al., 2023 [36]	Secondary analysis from C-RCT	Australia	• 269 Australian office workers • aged ≥ 18 years, • work ≥ 30 h/week	Five specific exercises (resisted flexion, extension and reverse flys) targeting the neck/shoulder area,	• Work productivity, neck pain • Health related quality of life, self-efficacy, • Behavior change, • Muscular performance, • Exercise compliance	• Neck and shoulder pain ↓ in RT group • median quitting time—6 to 8 weeks • 30% quit in week six • RT volume of 11,000 kg and progressions of 1 to 2 times—clinically relevant pain ↓
Depreli et al., 2024 [31]	Parallel group randomized study	Türkiye	• 60 office workers with neck-shoulder pain • Shoulder protraction • BMI < 30	• Three times/day, 10 reps, three sets • IG1: office exercises involving neck range of motion, shrugs, wall push • IG2: stabilization exercise with therabands and dumbbells, progressive resistance • Perceived exertion: 5–6/10 • 12 weeks – 8 weeks ex, 4 weeks detraining	• Shoulder muscle strength (manual muscle tester) • Shoulder proprioception (isokinetic dynamometer) – reproduction of active and passive position • Vibration sense (vibrometer) • Upper extremity functional performance	• acromion-bed distance ↓ • ↑ trapezius, deltoid and serratus anterior muscle strength, latissimus, and pectoralis • Time dependent ↓ in the skeletal muscle strength with 4 weeks of detraining • ↑ proprioception and functional performance with stabilization exercises
Granacher et al., 2011 [34]	Controlled longitudinal intervention study	Switzerland	• 34 Swiss office workers • Sedentary work • Age 56 years	• Eight weeks • workplace, • squats and calf raise • Three times/day, five days/ week • 4sets, 15 reps/set • eight weeks of detraining	• Balance • Gait speed • Peak muscular force • Jump height	*After training* • ↑ balance, jump height and gait speed *After detraining* • Lower limb strength ↓
Heredia-Rizo et al., 2019 [29]	Two parts: (1) cross-sectional trial; (2) randomized controlled trial	Spain	• 40 for part (a) and 20 female office workers in part (b) • pain > 12 weeks, • worst pain < 24 h	• Eccentric training • 10 sessions, ∼25 to 30 min upper trapezius • 3 sets, 10 reps at 60% MVC, 8 reps at 70% MVC and 6 reps at 80% MVC	• worst pain < 24 h • Nordic Musculoskeletal Question (pain and discomfort) • Arm, neck, and shoulder disability • Manual/ cuff pressure algometer measured pain pressure threshold	• 75%—bilateral symptoms • Pain intensities and neck/shoulder disability ↓ • no differences in pain pressure threshold over the neck and forearm (− 2.6 kPa) • pain detection threshold ↓ 30% pain modulation • ↑ pain threshold • No relation between pain modulation and disability
Ho et al., 2024 [28]	Randomized controlled trial	Taiwan	• 43 recruited • 36 middle-aged • 40–60 years • sedentary female workers	• Two IG (HIIT &AT) and one control (CG) groups • HICT: 20–35 min, 2–3 rounds, 8 min/round; 4–5 min interest rest • Three times per week for eight weeks • 24 sessions	• Body composition: BIA • Muscular performance (hand grip, back strength and lower limb strength at 60º/s and 180º/s • Biochemistry markers (hs-CRP and lipid profile)	• Muscle mass ↑, knee extension 60°/s ↑ in HIIT • Body weight ↓ (Δ 0.5 kg) in AT compared to CON • ↔ biochemistry markers (hs-CRP, TC, and LDL-C) or IGF-1
Karatrantou et al., 2023 [32]	Non-randomized controlled study	Greece	• Fifty office workers (17 ♂, 33 ♀ females) • administrative office • Active	• 50–60 min/session • Three days/ week, in a 4-month • Body awareness (Yoga), and circuit strength training programs • Circuit RT: 3–4 rounds. 10–12 min/round, 7–8 mules, multimodal	• Health Indices: BMI, body fat, musculoskeletal discomfort • Physical fitness • Enjoyment	• Health indices ↑ ((p < 0.01; d = 0.16–0.37) • musculoskeletal pain ↓ post-training (d = 1.08 = 1.50) • ↑ flexibility of lower and upper limbs, ↑ static and dynamic balance • push-up test ↑ (Δ 33.9%) • No Δ in fitness • High levels of enjoyment
Lidegaard et al., 2013 [35]	Single-group longitudinal follow-up	Denmark	• nested in a larger randomized controlled trial • 30 ♀ office workers • Neck pain	• Ten weeks • IG: Elastic Band-based RT • CG: weekly email-based information on general health	• EMG of splenius and upper trapezius • bipolar surface EMG configuration • EMGgap	• training adherence = 86.8% • EMG gap: duration ↑ by 71%, frequency ↑ by 296% • EMG max by 578% and 242%, splenius muscle • ↑ relaxation and pain
Meiling et al., 2019 [18]	Pilot RCT study	Australia	• 40 sedentary office workers • > 55 years old • independently ambulant, • engaged in < 60 min of MVPA	• IG: 24-step Yang style Taichi, thrice weekly for 12 weeks, 45 min/session. TheraBand with distinct colors • 24-step Yang style Taichi alone for 12 weeks	• Measured six weeks and 12 weeks • Subject: stress, anxiety, and depression • Objective: 30-s CST, flexibility, handgrip, 2-Minute Walk Test	• No change in scores of balances and endurance • lower limb strength ↑ (MD = 4.15; SD = 3.41) at 6 weeks and ↑ at 12 weeks (MD = 4.05; SD = 3.24) • right upper-limb strength ↑ (MD = 3.50; SD = 5.28) at six weeks and ↑ at 12 weeks (MD = 0.55; SD = 3.32) • anxiety ↓ 12 weeks (M = -1.65) • No meaningful change in stress and depression • Pain ↓ (MD = − 0.45; SD = 1.10) in 12 weeks
Mulla et al., 2018 [17]	Parallel-group, RCT	Canada	• Office employees at the Ford Motor Company • Forty-three desk-based workers • With chronic diseases limiting PA excluded	• IG: 3 RT classes/week, 45 min/session • strengthening exercises for lower extremity • static squats and lungs • RPE—5 and 7 from a scale of 0 to 10, • CG: refrain from strenuous PA for 12 weeks	• Physical function – lower extremity • Mobility and strength • Pain and depression – self reported • Workability outcomes • Treadmill 6-min walk test	• ↑ lower limb functions and mobility • No significant difference in strength and workability
Nikander et al., 2006 [25]	RCT	Finland	• aged 25–55 yr • Office workers • Neck pain—constant and frequent	• IG group: specific neck, shoulder, and upper extremities static and dynamic strengthening ex • three series of 20 reps • 80% max isometric strength • 12 months • CG: aerobics/ recreational class	• submaximal bicycle ergometer test -VO2 max • MET quantification from a monthly recall questionnaire	• VO2max did not differ among groups • ↑ aerobic capacity associated with ↓ pain
Pedersen et al., 2009 [27]	C-RCT	Denmark	• 549 office workers • 12 offices in Denmark • H/o chronic diseases/ trauma	• RT performed with dumbbells—shoulder girdle, and isometric exercises for the muscles of the cervical spine • 2–3 sessions/week • 20 min/session • 2 to 3 sets with 10 to 15 repetitions • 70% to 80% of maximal force	• Weekly MET quantified PA (self-reported Questionnaire) • Maximal voluntary isometric muscle strength of the shoulder, handgrip and back – Bofors dynamometer • Submaximal ergometer test	• Shoulder elevator strength ↑ with interventions • No change in VO2max • SRT and APE ↓ SBP (∼6.4 mm Hg), fat% (∼2.2%), as well as right shoulder and back pain (∼30%) • Muscle strength and VO2 ↑ approximately 10% • No change in productivity
Rogers Banks et al., 2024 [15]	Experimental trial (RCT)	USA	• 24 office workers of the campus • Fee from diseases • 18–45 years	• CG: 3 h. sitting after breakfast • IG: 3 min RT breaks every 30 min of 3-h trial • Squats, high knees, calf raises	• Biochemical markers – glucose, insulin, and endothelin • Vascular function – gastrocnemius perfusion, upper and lower limb blood flow	• Popliteal shear rate and gastrocnemius perfusion ↑ with resistance exercises • Glucose ↑ (+ 5.0 ± 2.0 mg/dL) • Insulin increased for the first 30 min; however, it dropped in the next 30 min in insulin
Rogers Banks et al., 2024 [33]	Feasibility study	USA	• 29 workers (20 females) • 19 and 64 years, • free from CMD • Sitting time > 7 h/day	• Assigned to one of 5 interventions • performed four RT visits in varying frequencies • every 2 h. on days 1 and 2, every one-hour day 3 and 4 and every 30 min on 5 & 6	• Step count using accelerometer • Work productivity • Sleepiness	• Stepping time and number of STS ↑ during RT week • Low frequency (4-RT, 8 RT) was acceptable • Overall discomfort ↓ 20% in RT and ↑ 50% in SIT
Saeterbakken et al., 2020 [37]	Single-group cross-over trial	Norway	• Thirty-three office workers with neck pain	• Interrupted time series • 16-week • Control period – 8 weeks; followed by intervention – 8 weeks • 10 min • High intensity shoulder and neck RT program	• visual analogue scale—pain • Likert scale 0–100 – quality of life • Neck and shoulder – isometric strength	• No differences • ↓ pain by 25% and 43% • 10.6% ↑ in quality of life • No difference in strength
Ylinen et al., 2003 [26]	RCT	Finland	• 180 ♀ desk-based office workers • 25–35 years • With neck pain for > 6 months	• IG: RT and AT groups • Therabands – neck strengthening and stabilization exercises • Five sessions/week, 45 min/session • CG • 12 months	• Subjectively perceived pain -VAS • Neck and shoulder disability • Short depression inventory • Max isometric neck strength • grip strength test • submaximal bicycle ergometer test	• RT ↑ strength and ↓ pain better than AT and control • Analgesic use ↓ in both intervention groups

APE – All round physical exercise, AT – aerobic training, BMI – body mass index, CG – control group, C-RCT – cluster randomized controlled trial, CRP—HIIT – high intensity interval training, CST – chair sit-stand test, EMG – electromyography, IG – intervention group, MD – mean difference, MET – metabolic equivalent, MVC – maximal voluntary contraction, RCT – randomized controlled trial, RM – repetition maximum, RPE – rate of perceived exertion, RT – resistance training, SD – standard deviation, SRT – specific resistance training, STS – sit-stand, VAS – visual analogue scale, VO2 max – maximal oxygen consumption

### Risk of Bias in the Included Studies

**Fig. 2:**
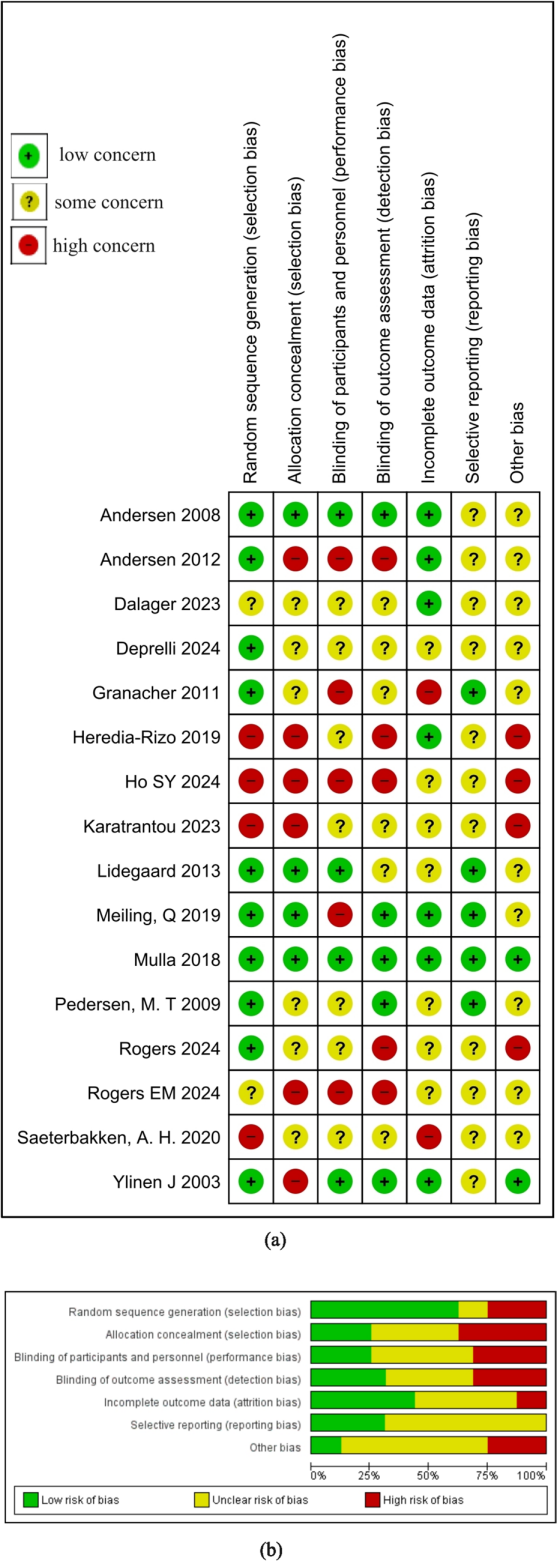
Risk of bias in the included studies. **a** risk of bias in the individual studies; **b** risk of bias summary

Only a few studies scored ‘low risk’ in each of the five domains (selection, performance, detection, attrition and reporting bias) [17, 18, 26, 30, 35]. Only a few studies have clearly mentioned the randomization method [17, 31] and have explicitly stated allocation concealment procedures[17, 18, 30, 35]. While few trials blinded their outcome assessor [17, 27, 30, 35], the statisticians and the therapists administered the interventions were seldom blinded [17, 30]. Only two trials have attempted to control behavioral contamination within the study participants, standardization of diet and baseline activity, uncertainty of effects and generalizability [17, 26]. Figure 2(a) and Fig. 2(b) show the risk of bias in the individual studies and summary of the risk of bias, respectively.

### Narrative Synthesis

#### Population

A total of 2,607 office workers from 17 studies participated, with a mean age of 44 years and an average body mass index (BMI) of 24.74 kg/m^2^. The majority of the study participants were female office workers (n = 1884, 72%). Few studies explicitly stated whether the office workers were sedentary [15, 28, 33, 34] or physically inactive [18] at the baseline. Most studies explored the effectiveness of RT programs among office workers with musculoskeletal (shoulder, back and neck) pain [16, 25, 26, 29, 30, 35, 37]. All the studies involved young—middle-aged sedentary office workers except two studies which involved middle-old sedentary office workers with the mean age of 55 years and above [18, 34]. Few studies have explored the intervention’s effect on female office workers [25, 29, 35].

#### Intervention

The majority of the included studies administered RT programs specific to neck and shoulder strength at natural workplace setting without traditional gym-based training using body weight, dumbbells and therabands [15, 18, 26, 27, 30, 31, 33, 35–37], while few administered gym-based training (MVC and 1-RM) in the workplace [29] and few engaged in a gym setting outside workplace [25, 28, 32]. A cross-over study by Rogers et al., 2024 examined the acceptability of three-minute RT breaks (‘exercise snacks’) at varied frequencies (every 30 min, one hour, and two hours) [33]. Target muscles were neck and shoulder girdle (trapezius, cervical muscles) using therabands, medicine balls, body weight, dumbbells and TRX [31, 32]. Circuit training with 3–4 rounds, 10–12 min/round and 7–8 polyarticular muscles per day, thrice per day, five days per week, four sets, 15 reps/set was followed [28, 32, 34]. Only a study by Heredia-Rizo et al. 2019 described the dose and progression appropriately: three sets, ten reps/set at 60% MVC to 6 reps at 80% MVC [29]. However, none of the studies advocated a traditional progressive RT program that involved 1-RM determination using machine plates and the progression in office workers either in the space within or outside workplaces.

#### Outcomes

Most of the included studies focused on reducing musculoskeletal pain and discomfort [16, 18, 25, 26, 29, 30, 32, 35–37]. The included studies rarely evaluated the muscle strength of hand, shoulder and back [27], lower extremity isokinetic strength [28, 34], sub maximal aerobic capacity [17, 25], physical capacity – gait speed, walk and stair climb [17, 18, 34], physical fitness [32], physical activity levels [27, 33], work productivity [33, 36], sleepiness [33], psychological outcomes such as anxiety and depression [17, 18], workability and productivity [17], serum lipids and inflammatory markers—CRP [28] and finally behavior change [36]. Only one study explored occupation-related muscle activation using electromyography [35]. Recent studies have explored the proprioception and functional performance changes with the resistance exercises among office workers which are crucial for long-term postural control and the musculoskeletal outcomes [31].

#### Key Findings (effectiveness of RT programs among desk-based office workers)

Muscle strength (neck, shoulder) significantly improved by 6% – 13% in the majority of the studies [16, 18, 28, 30, 32] but not balance and endurance [18]. Mulla demonstrated no significant change in knee strength and work-related outcomes, with improvements in lower extremity function and mobility evident after 12 weeks of RT among desk-based office workers [17]. Deprelli et al., 2024 found that 8 weeks of specific scapular stabilization exercises along with traditional neck exercises improve neck and shoulder strength, proprioception, joint sense and may remain higher than baseline at 12th week even after the intervention ceases [31]. Rogers et al., 2024 demonstrated a significant improvement in stepping time, sit-stand transitions and prolonged sedentary bout > 30 min, activity score, total sedentary time and daily step count remain unchanged with the RT program [33]. While few studies found a reduction in neck and shoulder pain [29, 30, 36] with increased pain threshold [29], few studies did not observe any significant difference in neck pain compared to control and baseline values [16]. No change in lipid or inflammatory markers, especially CRP and IGF [28] or depression [18] with the RT programs in workplaces. Physical performance in terms of gait speed isokinetic torque was found to be improved after RT at the workplace [34]. Adherence was found to be 87% with low-intensity RT programs in workplaces. However, adherence drops in 6th week, with 30% quitting [36]. While several studies explicitly stated that no adverse events associated with resistance training (RT) programs among office workers [17, 18, 29, 32, 33, 35], a cluster randomized controlled trial by Andersen et al. (2008) noted post-intervention discomfort in the shoulder, neck, and back, attributed to overexertion or incorrect strength training techniques [30].

## Meta-Analysis Findings

The meta-analysis of the outcome measures included in the pooled studies is illustrated in Table 3 below. Due to the paucity of the studies, the meta-analysis was administered for musculoskeletal outcomes (neck & shoulder discomfort & disability, musculoskeletal strength), metabolic outcomes (body fat, waist-hip ratio, BMI, and systolic pressure), aerobic capacity and static balance. The rest of the outcomes were explored by a lesser number of studies (n = 1) making the pooling of data difficult.

**Table 3:** The table demonstrates the pooled effects of resistance training on eight outcomes and their composite variables. Further strength of evidence is assessed using GRADE synthesis

Outcomes	Participants	No. of studies (design)	Effect size/risk	95% confidence intervals	Certainty of evidence
***Musculoskeletal pain/discomfort***	**2270**	**9 (mixed)**	**SMD 1.97 lower**	**4.4 lower to 0.45 higher**	⨁◯◯◯ **Very low**^**a,b,c,d,e**^
1. Neck discomfort	423	5 (mixed)	SMD 1.76 lower	2.46 lower to 1.06 lower	⨁◯◯◯ Very low^b,c,d,e^
2. Shoulder discomfort	631	2 (RCTs)	SMD 13.29 lower	15.87 lower to 10.71 lower	⨁◯◯◯ Very low^a,b,c,d,e^
3. Pain sensitivity threshold	80	2 (non-RCTs)	SMD 5.81 lower	13.85 lower to 2.23 higher	⨁◯◯◯ Very low^a,b,c,d,e^
***Musculoskeletal strength***	**1651**	**8 (RCTs)**	**SMD 2.73 higher**	**2.56 higher to 2.91 higher**	⨁⨁◯◯ **Low**^**a,b,c,d,e**^
1. Back extensor strength	446	3 (RCTs)	SMD 2.80 higher	0.02 higher to 5.94 higher	⨁◯◯◯ Very low^a,b,c,d,e^
2. Neck extensor strength	210	3 (RCT)	SMD 9.07 higher	8.06 higher to 10.07 higher	⨁⨁◯◯ Low^a,b,c,d,e^
3. Shoulder strength	466	3 (RCTs)	SMD 4.13 higher	0.42 higher to 7.84 higher	⨁⨁◯◯ Low^a,b,c,d,e^
4. Hand grip	107	3 (RCTs)	SMD 4.62 higher	1.89 higher to 7.35 higher	⨁⨁◯◯ Low^a,b,c,d,e^
***Neck-shoulder-Arm-Leg perceived ability***	**213**	**3 (RCTs)**	**SMD 0.31 higher**	**2.02 lower to 2.64 higher**	⨁⨁◯◯ **Low**^**a,b,c,d,e**^
***Metabolic risk outcomes***	**1258**	**4 (mixed)**	**SMD 3.45 lower**	**5.56 lower to 1.34 lower**	⨁◯◯◯ **Very low**^**a,b,c,d,e**^
1. Body fat percentage	436	3 (2 RCTs and one non-RCT)	SMD 8.76 lower	12.58 lower to 4.94 lower	⨁◯◯◯ Very low^a,b,c,d,e^
2. Waist-hip ratio	74	2 (RCTs)	SMD 0.01 higher	0.44 lower to 0.47 higher	⨁◯◯◯ Very low^a,b,c,d,e^
3. BMI	74	2 (RCTs)	SMD 4.14 lower	13.07 lower to 4.8 higher	⨁◯◯◯ Very low^a,b,c,d,e^
4. Systolic blood pressure	410	2 (non-RCTs)	SMD 11.34 lower	60.77 lower to 38.08 lower	⨁◯◯◯ Very low^a,b,c,d,e^
***Aerobic capacity***	**74**	**2 (RCTs)**	**SMD 0.42 higher**	**0.01 lower to 0.86 higher**	⨁⨁◯◯ **Low**^**a,b,c,d,e**^
***Balance***	**90**	**2 (RCTs)**	**SMD 22.2 higher**	**21.22 lower to 65.95 higher**	⨁⨁◯◯ **Low**^**a,b,c,d,e**^

Bold font signifies main domains depicting health outcomes

BMI – body mass index, EMG – electromyograph, ET – endothelin, hs-CRP – high sensitive C-Reactive Protein, IGF – insulin like growth factor, LDL-C – low density lipoprotein cholesterol, MD – mean differences, SMD – standardized mean differences; Certainty of evidence: ⨁⨁⨁⨁—High, ⨁⨁⨁◯—Moderate, ⨁⨁◯◯—Moderate, ⨁◯◯◯—Very low (assessed using GRADE synthesis) ^a^serious risk of bias; ^b^inconsistency; ^c^serious indirectedness; ^d^serious imprecision; ^e^strongly suspected publication bias

### Musculoskeletal Outcomes

Musculoskeletal Discomfort.

**Fig. 3:**
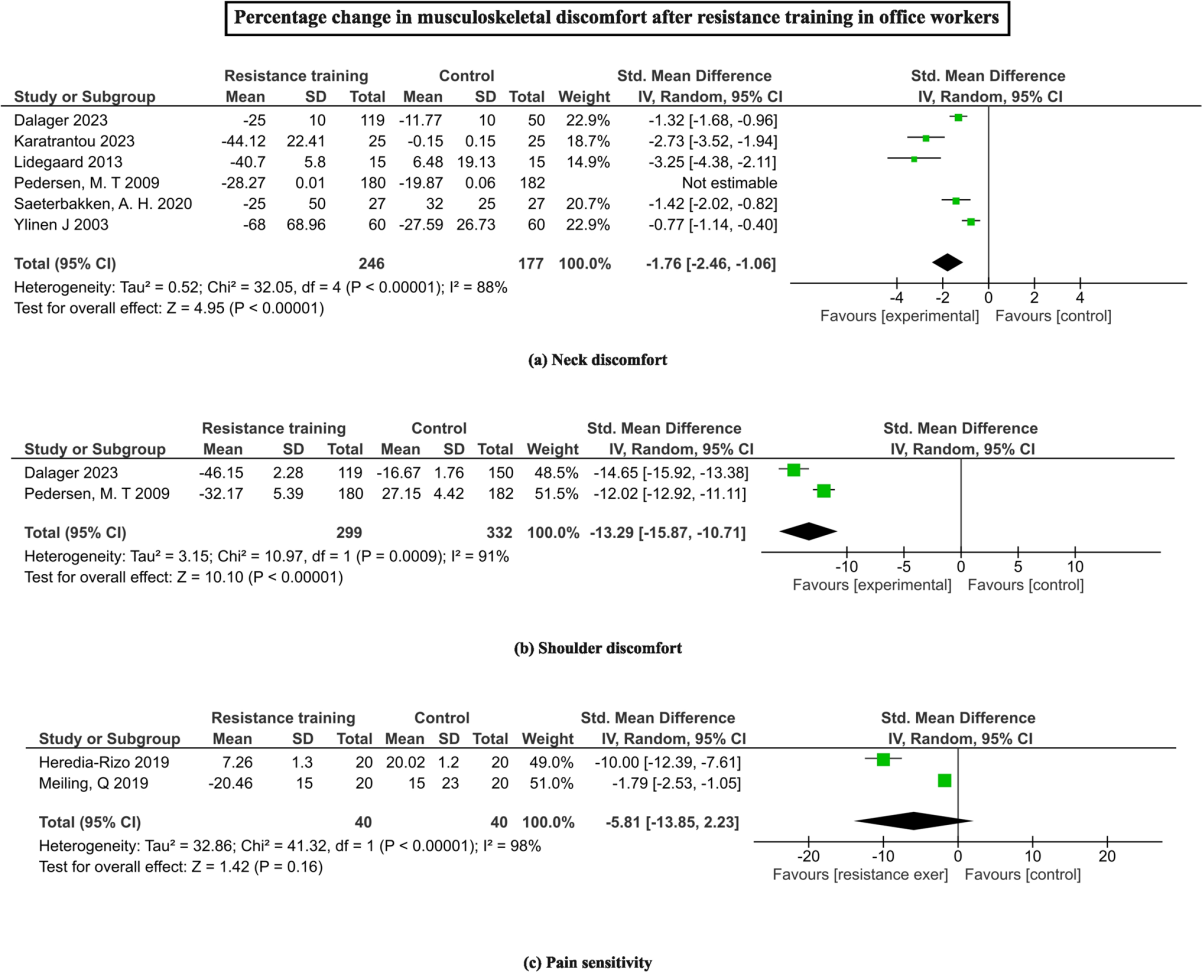
Forrest plots depicting the pooled means of change in muscle discomfort **a** neck discomfort, **b** shoulder discomfort, **c** pain sensitivity with the resistance training among office workers

Figure 3 demonstrates the changes in musculoskeletal discomfort with RT programs among office workers. High heterogeneity was observed in all the studies in outcome measurement and intervention administration. A significant larger reduction in shoulder discomfort (SMD = -13.29, 95% CI = -15.87 to -10.71; I^2^ = 91%, Z = 10.10, p < 0.00001) from two studies [27, 36] (Fig. 3b). From six studies [26, 27, 32, 35–37], neck discomfort was found to significantly reduced with the RT programs (SMD = -1.76, 95% CI = -2.46 to -1.06; I^2^ = 88%, Z = 4.95, p < 0.00001) (Fig. 3a). RT programs involving eccentric training [29] or Taichi [18] showed non-significant larger reductions in the pain sensitivity (SMD = -5.81, 95% CI = -13.85 to -2.23; I^2^ = 98%, Z = 1.42, p = 0.16) (Fig. 3c).

Musculoskeletal Strength.

**Fig. 4:**
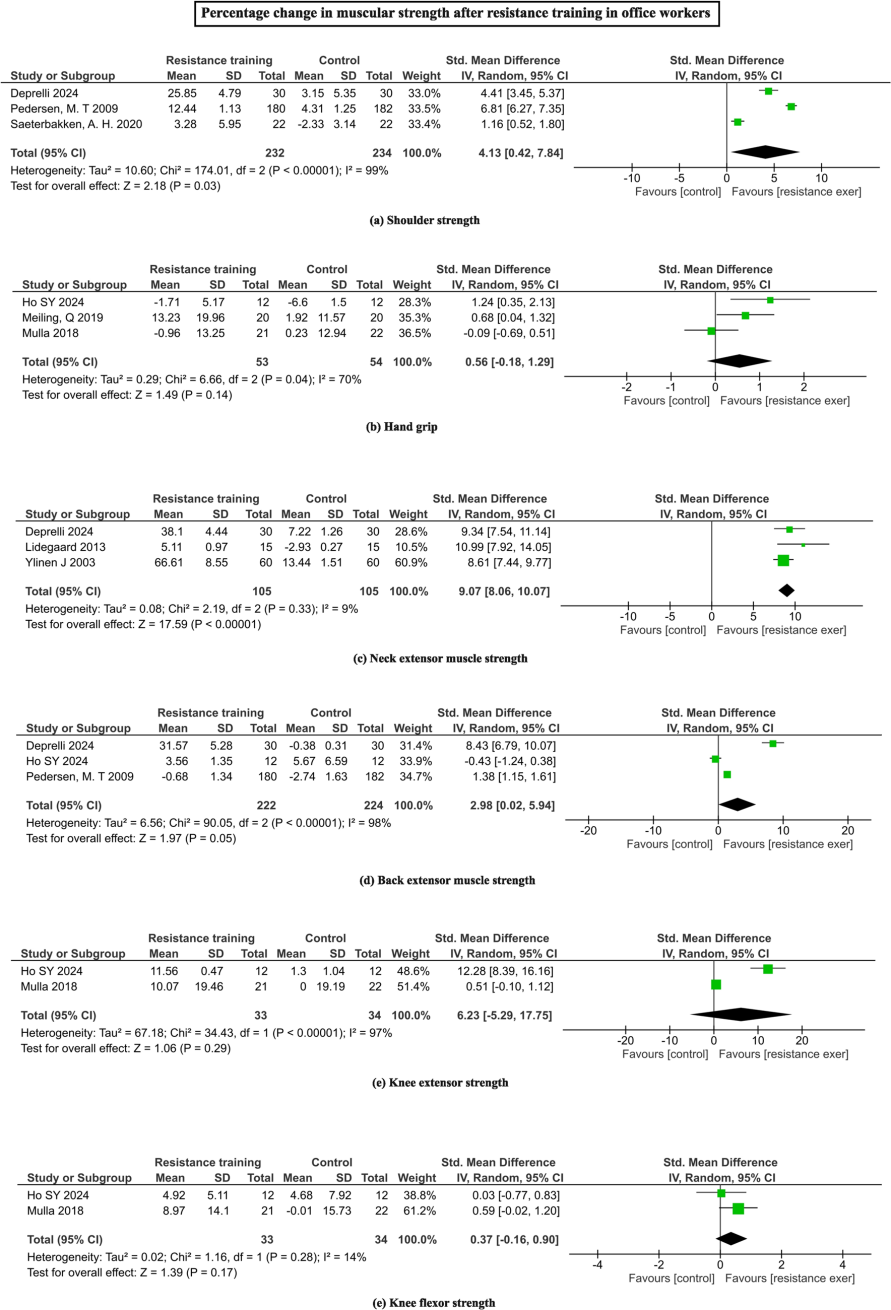
Forrest plots depicting the pooled means of muscle strength **a** shoulder strength, **b** handgrip, **c** neck extensor, **d** back extensor, **e** knee extensor, **f** knee flexor strength changes with the resistance training among office workers

Pooled analyses showed that RT leads to significant larger improvement in overall musculoskeletal strength (SMD = 2.73, 95% CI = 2.56 to 2.91; I^2^ = 99%, Z = 30.71, p < 0.00001). When analyzed the individual regions, only neck extensor muscle strength (SMD = 9.07, 95% CI = 8.06 to 10.07; I^2^ = 9%, Z = 17.59, p < 0.00001), shoulder muscle strength (SMD = 4.13, 95% CI = 0.42 to 7.84; I^2^ = 99%, Z = 2.18, p = 0.03) and back extensor (SMD = 2.98, 95% CI = 0.02 to 5.94; I^2^ = 98%, Z = 1.97, p = 0.05) showed statistically significant larger increase with RT programs, while improvements in the knee extensor muscle strength (SMD = 6.23, 95% CI = -5.29 to 17.75; I^2^ = 100%, Z = 1.06, p = 0.29), knee flexor strength (SMD = 0.37, 95% CI = -0.16 to 0.90; I^2^ = 14%, Z = 1.39, p = 0.17) and handgrip (SMD = 0.56, 95% CI = -0.18 to 1.29; I^2^ = 70%, Z = 1.49, p = 0.14) were not statistically significant. Figure 4 depicts the changes in regional muscular strength to RT programs.

Balance.

**Fig. 5:**
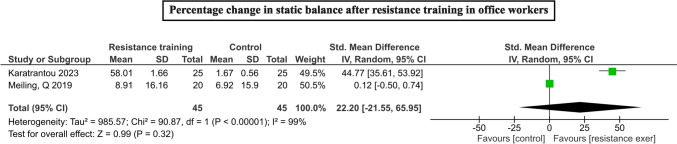
Forrest plots depicting the pooled means of change in static balance with the resistance training among office workers

On pooling of two studies [18, 32], RT group showed non-significant larger increase in the static balance especially single leg standing (SMD = 22.20, 95% CI = -21.55 to 65.95; I^2^ = 99%, Z = 0.99, p < 0.32). Figure 5 depicts the effect estimate of static balance with RT programs.

### Metabolic Outcomes

**Fig. 6:**
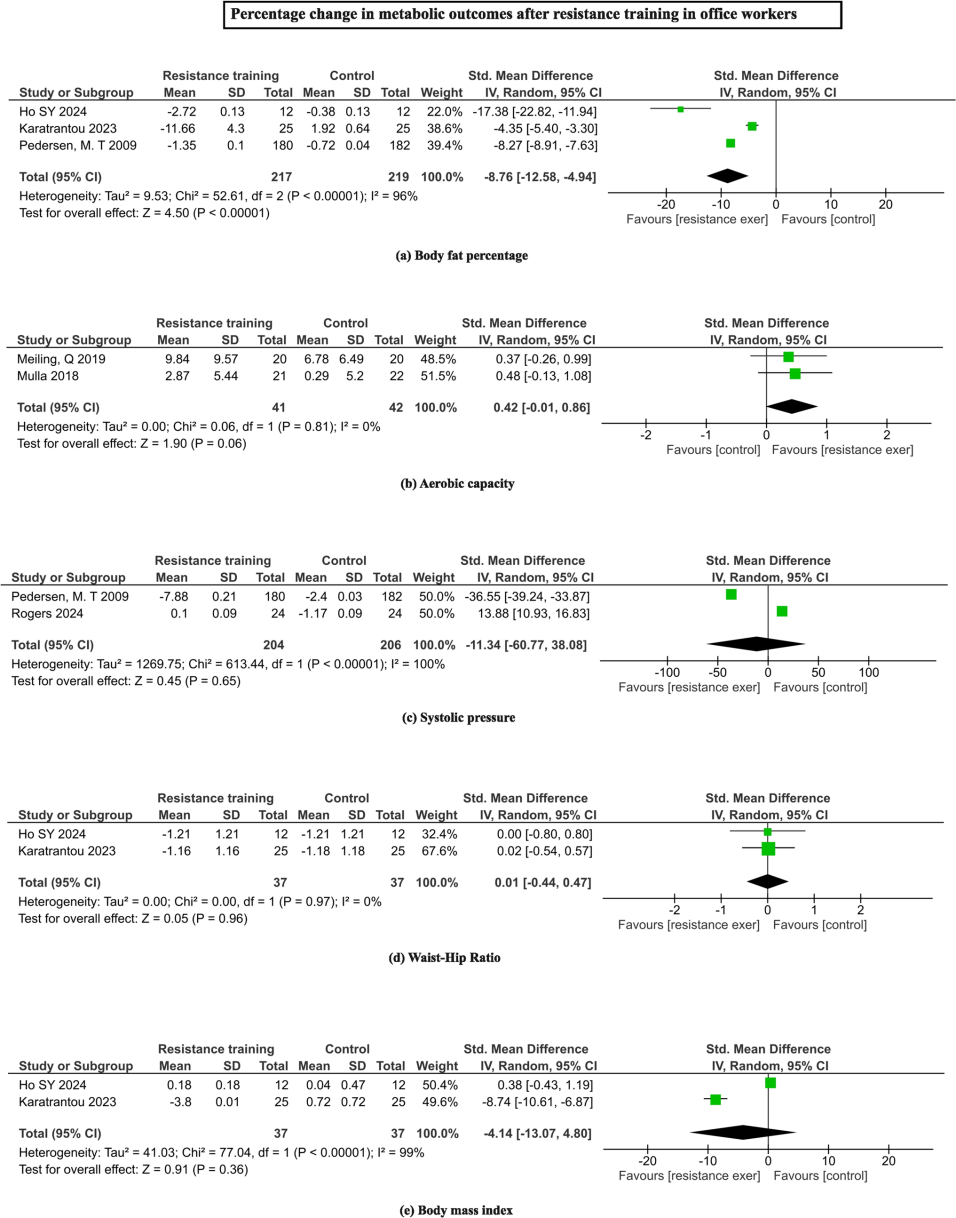
Forrest plots depicting the pooled means of metabolic outcomes **a** body fat percentage, **b** aerobic capacity, **c** systolic blood pressure, **d** waist-hip ratio, **e** body mass index changes with the resistance training among office workers

Of the four studies comprising 1258 participants, clustered metabolic risk factors were found to be significantly reduced with RT interventions (SMD = -3.45%, 95% CI = -5.56 to -1.34; I^2^ = 99%, Z = 3.2, p < 0.00001). When individual risk factors were analyzed independently, marginal reduction in fat percentages (SMD = -8.76, 95% CI = -12.58 to -4.94; I^2^ = 96%, Z = 4.50, p < 0.00001) were found, while body mass index (SMD = -4.14, 95% CI = -13.07 to 4.80; I^2^ = 99%, Z = 0.91, p = 0.36) did not show any statistical change. While aerobic capacity and waist-hip ratio remained unaltered, non-significant larger reductions in systolic pressure were observed with RT interventions in office workers (SMD = -11.34, 95% CI = -60.77 to 38.08; I^2^ = 100%, Z = 0.45, p = 0.65). Figure 6 depicts the pooling of metabolic risk parameters with RT interventions.

## Certainty of the Evidence

The included studies demonstrated: (1) high risk of bias (74%); (2) high heterogeneity (I^2^ > 75%)—inconsistency; (3) majority of the included studies included had only one outcome (imprecision); (4) no studies have clear eligibility criteria for generalization and potent confounders influencing results (indirectness). As only 2–3 studies were available for meta-analyses, publication bias was not assessed. To summarize, pooled analysis revealed very low certainty (strength) of evidence (Table 3) which meant further research in this area is very likely to have an important impact on confidence in estimate of effect and is likely to change the estimate.

## Discussion

### Summary of our Review Findings

Our systematic review aimed to explore the independent effects of resistance training on musculoskeletal and metabolic health outcomes in office workers during work and non-work hours. All the 17 studies which were included demonstrated heterogenicity in setting, intervention (dose) and outcomes. We found only a few studies were available to pool the data. Modest effect was evident in muscular discomfort with marginal reduction in the neck, back discomfort and pain sensitivity. While marginal improvement in back extensor, neck extensor, shoulder and hand grip strength observed, there is no change in the metabolic risk variables (body fat percentage, waist hip ratio, BMI, aerobic capacity, and systolic BP) with RT programs. The protective effects of RT programs on oxidative stress, inflammatory markers, vascular functions, mental health outcomes and workability are still emerging. Further emerging studies started employing novel objective measurements such as electromyography, doppler and accelerometers. Our review findings demonstrated that the RT administered as continuous or intermittent bouts were found to improve muscle strength, reduce muscular discomfort with no changes in cardiometabolic health risk among office workers. However, the certainty of the evidence was found to be very low due to high risk of bias, highly inconsistent, imprecise and exhibited high publication bias making the results less generalizable. Our findings should be interpreted with caution, as the effect estimates are somewhat uncertain. Future research is highly likely to significantly influence our confidence in these estimates and may lead to substantial changes in the results.

## Comparison with Previous Systematic Reviews

Recent systematic reviews have explored the efficacy of workplace physical activity interventions on neck pain [23, 38–40], low backpain [41, 42], wellbeing [43], sedentary time [44–47], cardiometabolic risk [9, 10, 48], mental health outcomes [49–51], work productivity [52] and health related quality of life [53]. Several types of interventions have been explored such as digital elements [54, 55], activity permissive workstations [46, 56], organizational polices and multicomponent interventions [45] to promote PA among office workers thereby improving health. As evident from the contemporary systematic reviews, studies exploring specific neck/shoulder strengthening exercises to improve strength and reduce neck/shoulder discomfort with computer work were included [39, 40]. However, an evidence summary claiming the isolated efficacy of RT programs on cardiometabolic and mental health benefits in office workers are lacking. To our knowledge there is only one recent narrative review has provided recommendations for RT (weekend warrior, exercise snacks, single set) and benefits in general population [57]. The larger effects in muscular strength and discomfort were observed in the studies which administered RT programs under supervision demonstrated by previous systematic reviews [42].

Our review findings align with recent systematic reviews that highlight the modest reduction in the muscle discomfort with RT interventions [38–40]. Majority of the studies have administered shoulder and neck specific RT programs making difficult to generalize RT recommendations for office workers. Our review findings depict that regular engagement in RT reduces nociception, despite the varying doses administered in the included studies. Also, our findings on marginal improvement in muscular strength concurs with the recent systematic review on systematic reviews [57, 58]. The potential reasons for only modest improvement in muscular strength may be due to the low load, or non-specific exercises and heterogeneity in the strength measurement administered in the workplace studies. The RT interventions were administered for varying mode (gym based and workplace based, therabands, eccentric training, circuit training) rarely addressing intensity (60–80% MVC [29]). Future studies should explore the dose–response relation of strengthening exercise on muscular strength in office workers. Static balance was found to be marginally improved with the RT program from two studies [18, 32]. Rt programs are speculated to reduce intracortical and corticospinal pathways while improving propagation velocity of the action potentials of the muscle fibers which eventually improves the static and dynamic balance of the individuals [59].

Our review findings conclude that RT programs offer lesser or no cardiometabolic or mental health benefits. Also, our review finds RT programs are being explored for their efficacy in inflammatory markers such as insulin like growth factor, C Reactive protein and endothelin in recent studies [28, 33]. In the near future, we hope these novel biomarkers will enhance our understanding of how RT programs can ameliorate cardiometabolic risk among office workers.

## Practice/research Implications

To date, no reviews have specifically focused on the pooled analysis of resistance exercise interventions alone for their potential health benefits among office workers. While research in this area is still in its early stages, we believe that resistance exercise regardless of intensity (e.g., body-supported, gym-based, or using free weights or elastic bands) hold considerable potential for improving health outcomes in sedentary workplaces. Furthermore, we contend that the current systematic review and meta-analysis, despite being based on limited and low-quality evidence, can serve as a catalyst for conducting high-quality randomized controlled trials. These future trials should aim to explore the health benefits of resistance exercise programs, whether through simple elastic bands or body-supported exercises, and to evaluate organizational policy changes that promote the adoption of gym facilities integrated into modern workplaces and daily routines of sedentary office workers. From the evidence, we can postulate the following recommendations for the RT for prevention or management of muscular discomfort and improving muscular strength among office workers. The significant balance and musculoskeletal benefits highlighted in our review findings may support and enhance existing policies aimed at incorporating RT programs to address musculoskeletal issues during retirement. A recommendation for the RT programs to improve health benefits based on the review findings can be found in Table 4.

**Table 4:** Recommended dosages for workplace RT programs to enhance health outcomes in office workers

RT dimensions	Dosage
*Frequency*	2–3 times/week (gym-based)[17, 27, 32], everyday (body weight support, dumbbell, elastic bands) as a 3 min bout every 30 min during working hours – ‘RT snacking’ [15, 33]
*Intensity*	20-RM to 8 RM[16, 25], 5–7 out of 0–10 RPE scale [17, 31], 80% of maximal isometric neck strength [25], 60% to 80% of MVC [27, 29, 30, 36]
*Duration*	20 min – 90 min[29]/session, 8–12 weeks (12 days to 12 months) [16, 17, 25, 26, 34]
*Phases*	10 min (warm up) – 30 min (exercise) – 5 min (cool down)[18, 32]
*Mode*	Gym-based training – supervised [17, 27, 32], neck flexor [26], eccentric training [29], body weight/ elastic band [18, 31, 35, 36] /free weight/ dumbbell [16, 25, 27] (vicinity of workstation)
*Volume*	8 – 15 reps/set, 2–3 sets/ muscle, eight larger muscles [30, 31, 34]
*Progression*	20 RM (initial weeks) to 8 RM (at 20th week)[16], dumb bell progressed from 2 to 13 kgs [25], reduction in sensory input [34]
*Type of exercises*	front raise, lateral raise, reverse flies, shrugs and wrist extension with dumbbells or elastic bands [16, 25, 27, 31, 35], shoulder girdle eccentric training[29, 30], bodyweight—squats, high knees, gluteal contractions, calf raises[15, 34], static squats, lunges [17], jumping jacks, wall sit, kneeing push-up, abdominal crunch, step-up, squat, triceps dip on chair, plank, high knee running in place, lunge, T rotation, and left/right side plank [28],

*MVC – maximal voluntary contraction, Reps – repetitions, RM – repetition maximum, RPE – rating of perceived exertion, RT – resistance training*

## Strength and Limitations

The strengths of this systematic review include: (1) its focus on evaluating the isolated effectiveness of RT programs specifically among sedentary office workers; (2) the use of rigorous search strategies across four electronic databases, coupled with risk of bias assessment conducted independently by multiple authors using Cochrane methodology and GRADE evidence synthesis; and (3) the pooling of studies to provide comprehensive summary findings that are directly applicable to occupational health practices. The limitations of our review are: (1) The protocol was not pre-registered in PROSPERO registry. At the time of initiating this review, we were not fully aware of the PROSPERO registration process or its requirements. By the time we learned about it, the review was already well underway, and it was too late to register prospectively. However, we have made every effort to ensure that the review process and results are reported transparently and comprehensively in accordance with PRISMA guidelines, which mitigates the concern of selective reporting or bias; (2) We included both randomized and non-randomized controlled trials, as the administration of RT programs for health benefits among office workers is limited. Additionally, we did not perform subgroup analyses for short-term (< 3 weeks) and long-term (> 3 weeks) trials due to the paucity of available evidence; (3) The large effects observed are likely influenced by significant publication bias, highlighting a lack of studies to provide balanced results. We recommend conducting high-quality trials with well-defined eligibility criteria, optimized RT program dosages, adherence monitoring, and standardization of confounding factors to strengthen the evidence on the efficacy of RT programs in improving health outcomes among office workers; (4) A high level of heterogeneity was observed across nearly all study outcomes, with only a limited number of studies evaluating these outcomes. Readers are advised to interpret the findings with caution, as this review included only a small number of studies; (5) The present review was limited to studies published in English, potentially excluding relevant studies available in gray literature or other languages. Additionally, as the search was confined to only four databases, future reviews should consider including a broader range of databases to ensure comprehensive coverage; and (6) All the included studies in the review were conducted in the office workers of high-income countries making it difficult to generalize in workspaces of low-middle income countries which differ in culture and organizational norms.

## Conclusions

Resistance exercises may offer a promising countermeasure for musculoskeletal discomfort and cardiometabolic risk among office workers, who are prone to high levels of sedentary behavior during working hours. However, the current evidence is weak and uncertain. Further high-quality trials are needed to strengthen evidence on the protective effects of RT programs against musculoskeletal and cardiometabolic health risks among office workers.

## Supplementary Information

Below is the link to the electronic supplementary material.

Supplementary file1 (DOCX 26 KB)

## Data Availability

No datasets were generated or analyzed during the current study.

## Abbreviations

APE: All round physical exercise
AT: Aerobic training
BMI: Body mass index
CG: Control group
C-RCT: Cluster randomized controlled trial
CRP: C-reactive protein
HIIT: High intensity interval training
CST: Chair sit-stand Test
EMG: Electromyography
IG: Intervention group
MD: Mean difference
MET: Metabolic equivalent
MVC: Maximal voluntary contraction
RCT: Randomized controlled trial
RPE: Rate of perceived exertion
RT: Resistance training
SD: Standard deviation
SRT: Specific resistance training
VAS: Visual analogue scale
*VO*_*2*_* max*: Maximal oxygen consumption

## References

[1] Martinez R, Lloyd-Sherlock P, Soliz P, Ebrahim S, Vega E, Ordunez P, et al. Trends in premature avertable mortality from non-communicable diseases for 195 countries and territories, 1990–2017: a population-based study. Lancet Glob Health. 2020;8(4):e511–23. 10.1016/S2214-109X(20)30035-8. 32199120 10.1016/S2214-109X(20)30035-8

[2] Uddin R, Lee EY, Khan SR, Tremblay MS, Khan A. Clustering of lifestyle risk factors for non-communicable diseases in 304,779 adolescents from 89 countries: a global perspective. Prev Med. 2020;131:105955. 10.1016/j.ypmed.2019.105955. 31862205 10.1016/j.ypmed.2019.105955

[3] Sarveswaran G, Kulothungan V, Mathur P. Clustering of noncommunicable disease risk factors among adults (18–69 years) in rural population. South-India Diabetes Metab Syndr. 2020;14(5):1005–14. 10.1016/j.dsx.2020.05.042. 32623362 10.1016/j.dsx.2020.05.042

[4] Tremblay MS, Aubert S, Barnes JD, Saunders TJ, Carson V, Latimer-Cheung AE, et al. Sedentary Behavior Research Network (SBRN) - Terminology Consensus Project process and outcome. Int J Behav Nutr Phys Act. 2017;14(1):75. 10.1186/s12966-017-0525-8. 28599680 10.1186/s12966-017-0525-8 PMC5466781

[5] Owen N, Sparling PB, Healy GN, Dunstan DW, Matthews CE. Sedentary behavior: emerging evidence for a new health risk. Mayo Clin Proc. 2010;85(12):1138–41. 10.4065/mcp.2010.0444. 21123641 10.4065/mcp.2010.0444 PMC2996155

[6] Kumar S. Theories of musculoskeletal injury causation. Ergonomics. 2001;44(1):17–47. 10.1080/00140130120716. 11214897 10.1080/00140130120716

[7] Koohsari MJ, Yasunaga A, McCormack GR, Shibata A, Ishii K, Nakaya T, et al. Domain-specific active and sedentary behaviors in relation to workers’ presenteeism and absenteeism. J Occup Environ Med. 2021;63(10):e685–8. 10.1097/JOM.0000000000002333. 34310542 10.1097/JOM.0000000000002333 PMC8478307

[8] Paluch AE, Boyer WR, Franklin BA, Laddu D, Lobelo F, Lee DC, et al. Resistance exercise training in individuals with and without cardiovascular disease: 2023 update: a scientific statement from the american heart association. Circulation. 2024;149(3):e217–31. 10.1161/CIR.0000000000001189. 38059362 10.1161/CIR.0000000000001189 PMC11209834

[9] Reed JL, Prince SA, Elliott CG, Mullen KA, Tulloch HE, Hiremath S, et al. Impact of workplace physical activity interventions on physical activity and cardiometabolic health among working-age women: a systematic review and meta-analysis. Circ Cardiovasc Qual Outcomes. 2017;10(2):e003516. 10.1161/CIRCOUTCOMES.116.003516. 10.1161/CIRCOUTCOMES.116.003516 28228457

[10] Mulchandani R, Chandrasekaran AM, Shivashankar R, Kondal D, Agrawal A, Panniyammakal J, et al. Effect of workplace physical activity interventions on the cardio-metabolic health of working adults: systematic review and meta-analysis. Int J Behav Nutr Phys Act. 2019;16(1):134. 10.1186/s12966-019-0896-0. 31856826 10.1186/s12966-019-0896-0 PMC6923867

[11] Brierley ML, Chater AM, Smith LR, Bailey DP. The effectiveness of sedentary behaviour reduction workplace interventions on cardiometabolic risk markers: a systematic review. Sports Med. 2019;49(11):1739–67. 10.1007/s40279-019-01168-9. 31429035 10.1007/s40279-019-01168-9

[12] Burn NL, Weston M, Maguire N, Atkinson G, Weston KL. Effects of workplace-based physical activity interventions on cardiorespiratory fitness: a systematic review and meta-analysis of controlled trials. Sports Med. 2019;49(8):1255–74. 10.1007/s40279-019-01125-6. 31115827 10.1007/s40279-019-01125-6

[13] Oye-Somefun A, Azizi Z, Ardern CI, Rotondi MA. A systematic review and meta-analysis of the effect of treadmill desks on energy expenditure, sitting time and cardiometabolic health in adults. BMC Public Health. 2021;21(1):2082. 10.1186/s12889-021-12094-9. 34774020 10.1186/s12889-021-12094-9 PMC8590128

[14] Ciolac EG, Rodrigues-da-Silva JM. Resistance training as a tool for preventing and treating musculoskeletal disorders. Sports Med. 2016;46(9):1239–48. 10.1007/s40279-016-0507-z. 26914266 10.1007/s40279-016-0507-z

[15] Rogers EM, Banks NF, Trachta ER, Wolf MS, Berry AC, Stanhewicz AE, et al. Resistance exercise breaks during prolonged sitting augment the blood flow response to a subsequent oral glucose load in sedentary adults. Exp Physiol. 2024. 10.1113/EP091535. 39093318 10.1113/EP091535 PMC13238634

[16] Andersen CH, Andersen LL, Gram B, Pedersen MT, Mortensen OS, Zebis MK, et al. Influence of frequency and duration of strength training for effective management of neck and shoulder pain: a randomised controlled trial. Br J Sports Med. 2012;46(14):1004–10. 10.1136/bjsports-2011-090813. 22753863 10.1136/bjsports-2011-090813 PMC3596862

[17] Mulla DM, Wiebenga EG, Chopp-Hurley JN, Kaip L, Jarvis RS, Stephens A, et al. The effects of lower extremity strengthening delivered in the workplace on physical function and work-related outcomes among desk-based workers: a randomized controlled trial. J Occup Environ Med. 2018;60(11):1005–14. 10.1097/JOM.0000000000001408. 30020219 10.1097/JOM.0000000000001408

[18] Meiling Q, Moyle W, Jones C, Weeks B. Effects of Tai Chi combined with theraband training on physical fitness, psychological well-being, and pain in older sedentary office workers: a pilot randomized controlled trial. Topics in Geriatric Rehabilitation. 2019;35(4):255–65. 10.1097/TGR.0000000000000244.

[19] Brandt T, Schwandner CTL, Schmidt A. Resistance exercise snacks improve muscle mass in female university employees: a prospective, controlled, intervention pilot-study. Front Public Health. 2024;12:1347825. 10.3389/fpubh.2024.1347825. 38379679 10.3389/fpubh.2024.1347825 PMC10877054

[20] Page MJ, McKenzie JE, Bossuyt PM, Boutron I, Hoffmann TC, Mulrow CD, et al. The PRISMA 2020 statement: an updated guideline for reporting systematic reviews. J Clin Epidemiol. 2021;134:178–89. 10.1016/j.jclinepi.2021.03.001. 33789819 10.1016/j.jclinepi.2021.03.001

[21] Liberati A, Altman DG, Tetzlaff J, Mulrow C, Gøtzsche PC, Ioannidis JP, et al. The PRISMA statement for reporting systematic reviews and meta-analyses of studies that evaluate health care interventions: explanation and elaboration. J Clin Epidemiol. 2009;62(10):e1-34. 10.1016/j.jclinepi.2009.06.006. 19631507 10.1016/j.jclinepi.2009.06.006

[22] Deeks JJ, Higgins JP, Altman DG. Analysing Data and Undertaking Meta-Analyses. Cochrane Handbook for Systematic Reviews of Interventions. 2008. p. 243–96.

[23] Chen X, Coombes BK, Sjøgaard G, Jun D, O’Leary S, Johnston V. Workplace-based interventions for neck pain in office workers: systematic review and meta-analysis. Phys Ther. 2018;98(1):40–62. 10.1093/ptj/pzx101. 29088401 10.1093/ptj/pzx101

[24] Dalton JE, Bolen SD, Mascha EJ. Publication bias: the elephant in the review. Anesth Analg. 2016;123(4):812–3. 10.1213/ANE.0000000000001596. 27636569 10.1213/ANE.0000000000001596 PMC5482177

[25] Nikander R, Mälkiä E, Parkkari J, Heinonen A, Starck H, Ylinen J. Dose-response relationship of specific training to reduce chronic neck pain and disability. Med Sci Sports Exerc. 2006;38(12):2068–74. 10.1249/01.mss.0000229105.16274.4b. 17146312 10.1249/01.mss.0000229105.16274.4b

[26] Ylinen J, Takala E, Nykänen M, Häkkinen A, Mälkiä E, Pohjolainen T, et al. Active neck muscle training in the treatment of chronic neck pain in women: a randomized controlled trial. JAMA: Journal of the American Medical Association. 2003;289(19):2509–16. 10.1001/jama.289.19.2509. 10.1001/jama.289.19.2509 12759322

[27] Pedersen MT, Blangsted AK, Andersen LL, Jørgensen MB, Hansen EA, Sjøgaard G. The effect of worksite physical activity intervention on physical capacity, health, and productivity: a 1-year randomized controlled trial. J Occup Environ Med. 2009;51(7):759–70. 10.1097/JOM.0b013e3181a8663a. 19528834 10.1097/JOM.0b013e3181a8663a

[28] Ho SY, Chung YC, Wu HJ, Ho CC, Chen HT. Effect of high intensity circuit training on muscle mass, muscular strength, and blood parameters in sedentary workers. PEERJ. 2024;12:e17140. 10.7717/peerj.17140. 10.7717/peerj.17140 PMC10962336 38529312

[29] Heredia-Rizo AM, Petersen KK, Madeleine P, Arendt-Nielsen L. Clinical outcomes and central pain mechanisms are improved after upper trapezius eccentric training in female computer users with chronic neck/shoulder pain. Clin J Pain. 2019;35(1):65–76. 10.1097/AJP.0000000000000656. 30222615 10.1097/AJP.0000000000000656

[30] Andersen LL, Jørgensen MB, Blangsted AK, Pedersen MT, Hansen EA, Sjøgaard G. A randomized controlled intervention trial to relieve and prevent neck/shoulder pain. Med Sci Sports Exerc. 2008;40(6):983–90. 10.1249/mss.0b013e3181676640. 18461010 10.1249/MSS.0b013e3181676640

[31] Depreli O, Erden Z. The effects of shoulder stabilization exercises on muscle strength, proprioceptive sensory ability and performance in office workers with shoulder protraction. Int J Occup Saf Ergon. 2024;30(2):599–610. 10.1080/10803548.2024.2326358. 38533585 10.1080/10803548.2024.2326358

[32] Karatrantou K, Batatolis C, Chatzigiannis P, Vasilopoulou T, Melissopoulou A, Ioakimidis P, et al. An enjoyable workplace combined exercise program for health promotion in trained employees: yoga, pilates, and circuit strength training. Sports. 2023;11(4):84. 10.3390/sports11040084. 10.3390/sports11040084 PMC10145485 37104158

[33] Rogers EM, Banks NF, Trachta ER, Barone Gibbs B, Carr LJ, Jenkins NDM. Acceptability of performing resistance exercise breaks in the workplace to break up prolonged sedentary time: a randomized control trial in U.S. office workers and students. Workplace Health and Safety. 2024;72(6):234–243. 10.1177/21650799231215814. 10.1177/21650799231215814 38314504

[34] Granacher U, Wick C, Rueck N, Esposito C, Roth R, Zahner L. Promoting balance and strength in the middle-aged workforce. Int J Sports Med. 2011;32(1):35–44. 10.1055/s-0030-1267214. 21072736 10.1055/s-0030-1267214

[35] Lidegaard M, Jensen RB, Andersen CH, Zebis MK, Colado JC, Wang Y, et al. Effect of brief daily resistance training on occupational neck/shoulder muscle activity in office workers with chronic pain: randomized controlled trial. BioMed Research International. 2013;2013:262386. 10.1155/2013/262386 PMC3892746 24490152

[36] Dalager T, Welch A, O’Leary SP, Johnston V, Sjøgaard G. Clinically relevant decreases in neck/shoulder pain among office workers are associated with strength training adherence and exercise compliance: explorative analyses from a randomized controlled trial. Phys Ther. 2023;103(2):pzac166. 10.1093/ptj/pzac166. 37104630 10.1093/ptj/pzac166

[37] Saeterbakken AH, Makrygiannis P, Stien N, Solstad TEJ, Shaw M, Andersen V, et al. Dose-response of resistance training for neck-and shoulder pain relief: a workplace intervention study. BMC Sports Science, Medicine and Rehabilitation. 2020;12:8. 10.1186/s13102-020-0158-. 10.1186/s13102-020-0158-0 PMC7110779 32266072

[38] Jones LB, Jadhakhan F, Falla D. The influence of exercise on pain, disability and quality of life in office workers with chronic neck pain: A systematic review and meta-analysis. Appl Ergon. 2024;117: 104216. 10.1016/j.apergo.2023.104216. 38219373 10.1016/j.apergo.2023.104216

[39] Louw S, Makwela S, Manas L, Meyer L, Terblanche D, Brink Y. Effectiveness of exercise in office workers with neck pain: a systematic review and meta-analysis. S Afr J Physiother. 2017;73(1):392. 10.4102/sajp.v73i1.392. 30135909 10.4102/sajp.v73i1.392 PMC6093121

[40] Frutiger M, Borotkanics R. Systematic review and meta-analysis suggest strength training and workplace modifications may reduce neck pain in office workers. Pain Pract. 2021;21(1):100–31. 10.1111/papr.12940. 32657531 10.1111/papr.12940

[41] Bell JA, Burnett A. Exercise for the primary, secondary and tertiary prevention of low back pain in the workplace: a systematic review. J Occup Rehabil. 2009;19(1):8–24. 10.1007/s10926-009-9164-5. 19219537 10.1007/s10926-009-9164-5

[42] Gobbo S, Bullo V, Bergamo M, Duregon F, Vendramin B, Battista F, et al. Physical exercise is confirmed to reduce low back pain symptoms in office workers: a systematic review of the evidence to improve best practices in the workplace. J Funct Morphol Kinesiol. 2019;4(3):43. 10.3390/jfmk4030043. 33467358 10.3390/jfmk4030043 PMC7739349

[43] Abdin S, Welch RK, Byron-Daniel J, Meyrick J. The effectiveness of physical activity interventions in improving well-being across office-based workplace settings: a systematic review. Public Health. 2018;160:70–6. 10.1016/j.puhe.2018.03.029. 29751224 10.1016/j.puhe.2018.03.029

[44] Wang C, Lu EY, Sun W, Chang JR, Tsang HWH. Effectiveness of interventions on sedentary behaviors in office workers: a systematic review and meta-analysis. Public Health. 2024;230:45–51. 10.1016/j.puhe.2024.02.013. 38503064 10.1016/j.puhe.2024.02.013

[45] Zhou L, Deng X, Guo K, Hou L, Hui X, Wu Y, et al. Effectiveness of multicomponent interventions in office-based workers to mitigate occupational sedentary behavior: systematic review and meta-analysis. JMIR Public Health Surveill. 2023;9: e44745. 10.2196/44745. 37494100 10.2196/44745 PMC10413238

[46] Neuhaus M, Eakin EG, Straker L, Owen N, Dunstan DW, Reid N, et al. Reducing occupational sedentary time: a systematic review and meta-analysis of evidence on activity-permissive workstations. Obes Rev. 2014;15(10):822–38. 10.1111/obr.12201. 25040784 10.1111/obr.12201

[47] Chu AH, Ng SH, Tan CS, Win AM, Koh D, Müller-Riemenschneider F. A systematic review and meta-analysis of workplace intervention strategies to reduce sedentary time in white-collar workers. Obes Rev. 2016;17(5):467–81. 10.1111/obr.12388. 26990220 10.1111/obr.12388

[48] Buffey AJ, Herring MP, Langley CK, Donnelly AE, Carson BP. The acute effects of interrupting prolonged sitting time in adults with standing and light-intensity walking on biomarkers of cardiometabolic health in adults: a systematic review and meta-analysis. Sports Med. 2022;52(8):1765–87. 10.1007/s40279-022-01649-4. 35147898 10.1007/s40279-022-01649-4 PMC9325803

[49] Chu AH, Koh D, Moy FM, Müller-Riemenschneider F. Do workplace physical activity interventions improve mental health outcomes? Occup Med (Lond). 2014;64(4):235–45. 10.1093/occmed/kqu045. 24850815 10.1093/occmed/kqu045

[50] Joyce S, Modini M, Christensen H, Mykletun A, Bryant R, Mitchell PB, et al. Workplace interventions for common mental disorders: a systematic meta-review. Psychol Med. 2016;46(4):683–97. 10.1017/S0033291715002408. 26620157 10.1017/S0033291715002408

[51] Martin A, Sanderson K, Cocker F. Meta-analysis of the effects of health promotion intervention in the workplace on depression and anxiety symptoms. Scand J Work Environ Health. 2009;35(1):7–18. 10.5271/sjweh.1295. 19065280 10.5271/sjweh.1295

[52] Marin-Farrona M, Wipfli B, Thosar SS, Colino E, Garcia-Unanue J, Gallardo L, et al. Effectiveness of worksite wellness programs based on physical activity to improve workers’ health and productivity: a systematic review. Syst Rev. 2023;12(1):87. 10.1186/s13643-023-02258-6. 37226273 10.1186/s13643-023-02258-6 PMC10207792

[53] Nguyen TM, Nguyen VH, Kim JH. Physical exercise and health-related quality of life in office workers: a systematic review and meta-analysis. Int J Environ Res Public Health. 2021;18(7):3791. 10.3390/ijerph18073791. 10.3390/ijerph18073791 PMC8038562 33916437

[54] Parés-Salomón I, Señé-Mir AM, Martín-Bozas F, Loef B, Coffey A, Dowd KP, et al. Effectiveness of workplace interventions with digital elements to reduce sedentary behaviours in office employees: a systematic review and meta-analysis. Int J Behav Nutr Phys Act. 2024;21(1):41. 10.1186/s12966-024-01595-6. 38641816 10.1186/s12966-024-01595-6 PMC11031993

[55] Taylor WC, Williams JR, Harris LE, Shegog R. Computer prompt software to reduce sedentary behavior and promote physical activity among desk-based workers: a systematic review. Hum Factors. 2023;65(5):891–908. 10.1177/00187208211034271. 34392738 10.1177/00187208211034271

[56] Silva H, Ramos PGF, Teno SC, Júdice PB. The impact of sit-stand desks on full-day and work-based sedentary behavior of office workers: a systematic review. Hum Factors. 2024. 10.1177/00187208241305591. 10.1177/00187208241305591 39626101

[57] Nuzzo JL, Pinto MD, Kirk BJC, Nosaka K. Resistance exercise minimal dose strategies for increasing muscle strength in the general population: an overview. Sports Med. 2024;54(5):1139–62. 10.1007/s40279-024-02009-0. 38509414 10.1007/s40279-024-02009-0 PMC11127831

[58] Rasmussen-Barr E, Halvorsen M, Bohman T, Boström C, Dedering Å, Kuster RP, et al. Summarizing the effects of different exercise types in chronic neck pain – a systematic review and meta-analysis of systematic reviews. BMC Musculoskelet Disord. 2023;24(1):806. 10.1186/s12891-023-06930-9. 37828488 10.1186/s12891-023-06930-9 PMC10568903

[59] Šarabon N, Kozinc Ž. Effects of resistance exercise on balance ability: systematic review and meta-analysis of randomized controlled trials. Life (Basel). 2020;10(11):284. 10.3390/life10110284. 10.3390/life10110284 PMC7697352 33203156

